# Holmium Laser Enucleation of the Prostate in a 400 cc Prostate: Case Report

**DOI:** 10.1089/cren.2015.0017

**Published:** 2016-02-01

**Authors:** Esha L. Gopee, Matthew K.H. Hong, Trung Pham

**Affiliations:** Department of Urology, Casey Hospital, Monash Health, Berwick, Australia.

## Abstract

The modality of choice in the surgical management of benign prostatic hyperplasia for large prostates has traditionally been open prostatectomy. Advances in minimally invasive techniques have begun to challenge this notion with advantages such as lower bleeding and transfusion rates and shorter hospital stay. In this case report, we illustrate the use of holmium laser enucleation of the prostate (HoLEP) in a gland measuring more than 400 cc. We describe the case of a 71-year-old man with persistent voiding urinary symptoms despite two previous transurethral resections of his prostate. With greater experience in HoLEP and declining experience in open prostatectomy, there may be a shift toward HoLEP as the preferred treatment choice for large prostate glands.

## Introduction

A number of endoscopic methods exist for the surgical management of benign prostatic hyperplasia (BPH), but large prostate glands have generally remained in the realms of open prostatectomy. The prostate gland can grow to remarkable sizes, with the term *giant prostatic hyperplasia* describing glands larger than 500 g,^1^ and the largest gland reported in the literature exceeding 700 g. Although these reports inevitably describe open enucleation of the prostate in their treatment, the increasing popularity and experience in holmium laser enucleation of the prostate (HoLEP) offers an endoscopic means to surgically address by enucleation such large prostate glands. Advantages of HoLEP over open prostatectomy include the ability to perform the procedure on selected anticoagulated patients and reduced intraoperative blood loss. Here, we report the case of a symptomatic 400 cc prostate gland enucleated by HoLEP. This is, to the best of our knowledge, the only HoLEP procedure reported on such a large prostate.

## Case Presentation

**FIG. 1.**
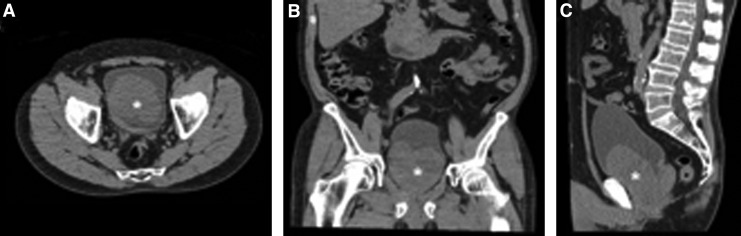
CT scan of the 10cm x 9cm x11cm prostate gland (*) in axial **(A)**, coronal **(B)** and sagittal **(C)** views demonstrating the large intravesical component.

A 71-year-old man first presented with severe hematuria 12 months before. He required transfusion with seven units of blood before resolution with conservative measures. He had undergone transurethral resection of the prostate (TURP) 8 and 15 years before, and his only regular medication was fish oil. Cystoscopy revealed massive regrowth of prostatic tissue with a large intravesical component. There was significant vascularity and contact bleeding was noted. CT excluded upper tract sources of hematuria and confirmed a markedly enlarged prostate measuring 10 × 9 × 11 cm (Fig. 1), and prostatic volume was estimated at 436 cc by ultrasonography of the prostate. His serum prostate-specific antigen was 17.9 ng/mL. Despite medical combination therapy of dutasteride and tamsulosin, during the next 12 months his urinary symptoms worsened and he continued to have intermittent hematuria. Uroflowmetry confirmed a poor voiding flow rate of 8.4 mL/s maximum flow and residual bladder volume of 400 mL, and we decided to proceed to HoLEP as an elective procedure.

During operation, the prostate was resected in 135 minutes using a 550-micron holmium:YAG laser fiber at 2 J × 50 Hz. Morcellation took 90 minutes by using the PIRANHA morcellation system (Richard Wolf GmbH, Knittlingen, Germany) with a total operating time of 225 minutes. The resected prostate tissue weighed 314 g. Postoperative catheterization time was about 35 hours and trial of void was effective with a residual bladder volume of 24 mL. There were no perioperative complications and blood transfusion was not necessary. Pre- and postprocedure hemoglobin levels were 135 and 150 g/L, respectively. The duration of inpatient stay was ∼53 hours.

Histopathologic examination of the prostatic tissue showed nodular hyperplasia without malignant change. At outpatient follow-up 2 months later, the patient reported good urinary flow with no further episodes of hematuria. Repeat uroflowmetry and ultrasonography showed a markedly improved maximum voiding flow rate of 28 mL/s with a voided volume of 584 mL and a residual bladder volume of 27 mL.

## Discussion

Endoscopic procedures are available for the surgical management of BPH, including TURP by monopolar or bipolar diathermy, laser photo vaporization of the prostate, HoLEP, and other minimally invasive methods such as thermotherapy and UroLift. Of these modalities, HoLEP is the only method that is not restricted by the size of a prostate gland.^2^

Our case highlights the feasibility of HoLEP in large prostates, where open prostatectomy might have previously been considered mandatory. Several studies in recent years have compared HoLEP with open prostatectomy. In one such randomized trial of 120 patients with prostates larger than 100 g, Kuntz and colleagues noted a significantly shorter catheterization time and length of inpatient stay, as well as a lower drop in mean hemoglobin for HoLEP than those for open prostatectomy.^3^ Eight patients who underwent open prostatectomy required a blood transfusion, whereas no patients required blood transfusion in the HoLEP group. During 18 months, there were no significant differences in complication rates and at 5-year follow-up, reoperation rates, maximum flow, and post-void residual were all similar.^4^ However, the main disadvantage of HoLEP was its greater duration of operation.

Another advantage of HoLEP over open prostatectomy is in the setting of anticoagulation. Whereas concurrent anticoagulation is contraindicated in TURP and open prostatectomy, the properties of laser have enabled prostatic procedures to occur on anticoagulated patients. In a retrospective study of patients undergoing HoLEP, Bishop and colleagues^5^ compared the outcomes of 52 patients operated under continuous antithrombotic therapy with outcomes of 73 patients not on antithrombotic therapy. Despite a predictably higher median length of stay and transfusion rate, the antithrombotic cohort did not require reintervention for bleeding. HoLEP seemed to be a safe procedure for patients with BPH who have an absolute clinical indication to remain on antithrombotic therapy. In their retrospective study of 79 patients undergoing HoLEP, Tyson and Lerner reported no difference in intraoperative hematuria and bleeding rates between those anticoagulated and those not anticoagulated, even after stratification for different anticoagulants.^6^ No patients required blood transfusion. This suggests that HoLEP may serve patients with large prostate sizes and medical comorbidities requiring continuous anticoagulation or antiplatelet therapies.

Although HoLEP has been performed since 1988,^7^ uptake in its technique has been relatively slow. An often cited obstacle is the perception of a steep learning curve compared with other urologic procedures. However, several studies now suggest that this is not the case. With thorough preparation, mentoring, and consolidation, morcellation efficiency can be achieved after 20 cases, and enucleation efficiency levels off after 30 cases. This contrasts with radical prostatectomy that appears to require a higher caseload to achieve acceptable competency.^8^

With the widespread availability of effective medical therapy and endoscopic surgery for BPH, large prostates are relatively rare and experience in open prostatectomy has declined immeasurably.^9^ Since HoLEP can be used on prostate glands of all sizes, experience in its use will naturally be more pervasive. Therefore, even large prostate glands could be addressed with HoLEP by urologists with experience gleaned from smaller prostates without the need for open prostatectomy.

## Learning Points

HoLEP is feasible for the surgical management of BPH in large prostates, with lower morbidity compared with open prostatectomy. Although such large prostate glands have traditionally been absolute indications for open prostatectomy, greater experience in HoLEP from smaller glands and more reports of HoLEP in ever-larger prostate glands suggest that the role of open prostatectomy will diminish.

## Disclosure Statement

No competing financial interests exist.

## Abbreviations Used

BPH: benign prostatic hyperplasia
CT: computed tomography
HoLEP: holmium laser enucleation of the prostate
TURP: transurethral resection of the prostate

## References

[1] Fishman JR, Merrill DC A case of giant prostatic hyperplasia. Urology 1993;42:336–337 7691015 10.1016/0090-4295(93)90628-n

[2] Vincent MW, Gilling PJ HoLEP has come of age. World J Urol 2015;33:487–493 25416347 10.1007/s00345-014-1443-x

[3] Kuntz RM, Lehrich K, Ahyai S Transurethral holmium laser enucleation of the prostate compared with transvesical open prostatectomy: 18-month follow-up of a randomized trial. J Endourol 2004;18:189–191 15072629 10.1089/089277904322959851

[4] Kuntz RM, Lehrich K, Ahyai SA Holmium laser enucleation of the prostate versus open prostatectomy for prostates greater than 100 grams: 5-year follow-up results of a randomised clinical trial. Eur Urol 2008;53:160–166 17869409 10.1016/j.eururo.2007.08.036

[5] Bishop C, Liddell H, Ischia J, Paul E, Appu S, Frydenberg M, Pham T Holmium laser enucleation of the prostate: Comparison of immediate postoperative outcomes in patients with and without antithrombotic therapy. Curr Urol 2013;7:28–33 24917753 10.1159/000343549 PMC3783280

[6] Tyson MD, Lerner LB Safety of holmium laser enucleation of the prostate in anticoagulated patients. J Endourol 2009;23:1343–1346 19575692 10.1089/end.2009.0013

[7] Fraundorfer MR, Gilling PJ Holmium:YAG laser enucleation of the prostate combined with mechanical morcellation: Preliminary results. Eur Urol 1998;33:69–72 9471043 10.1159/000019535

[8] van Rij S, Gilling PJ In 2013, holmium laser enucleation of the prostate (HoLEP) may be the new ‘gold standard’. Curr Urol Rep 2012;13:427–432 23054505 10.1007/s11934-012-0279-4

[9] Mearini E, Marzi M, Mearini L, Zucchi A, Porena M Open prostatectomy in benign prostatic hyperplasia: 10-year experience in Italy. Eur Urol 1998;34:480–485 9831789 10.1159/000019787

